# Malignancy risk of thyroid nodules: quality assessment of the thyroid ultrasound report

**DOI:** 10.1186/s12880-022-00789-3

**Published:** 2022-04-02

**Authors:** Luís Raposo, Cláudia Freitas, Raquel Martins, Catarina Saraiva, Isabel Manita, Maria João Oliveira, Ana Paula Marques, Bernardo Marques, Gustavo Rocha, Teresa Martins, Teresa Azevedo, Fernando Rodrigues

**Affiliations:** 1 Grupo de Estudo da Tiroide (GET), Sociedade Portuguesa de Endocrinologia, Diabetes e Metabolismo (SPEDM), Rua Fernando Vicente Mendes, 1B, 1600-892 Lisbon, Portugal; 2 Instituto de Saúde Pública, Universidade do Porto (ISPUP), Rua das Taipas 135, 4050-091 Porto, Portugal; 3 Hospital de Egas Moniz, Centro Hospitalar de Lisboa Ocidental, Rua da Junqueira, 126, 1349-019 Lisbon, Portugal; 4 Hospital de Santo António, Centro Hospitalar Universitário do Porto, Largo Prof. Abel Salazar, 4099-001 Porto, Portugal; 5 Instituto Português de Oncologia de Coimbra Francisco Gentil, Avenida Bissaya Barreto, 98, 3000-075 Coimbra, Portugal; 6 Hospital Garcia de Orta, Avenida Torrado da Silva, 2805-267 Almada, Portugal; 7 Centro Hospitalar de Vila Nova de Gaia/Espinho, Rua Conceição Fernandes, 4434-502 Vila Nova de Gaia, Portugal; 8 Hospital de Pedro Hispano, Unidade Local de Saúde de Matosinhos, Rua Dr. Eduardo Torres, 4464-513 Senhora da Hora, Porto, Matosinhos, Portugal; 9 Hospital Infante D. Pedro, Centro Hospitalar do Baixo Vouga, Avenida Artur Ravara, 3810-164 Aveiro, Portugal

**Keywords:** Thyroid, Thyroid nodules, Thyroid cancer, Ultrasound, Cancer risk, Clinical guidelines, Fine needle aspiration

## Abstract

**Background:**

Thyroid nodules are a challenge in clinical practice and thyroid ultrasonography is essential for assessing the risk of malignancy. The use of ultrasound-based malignancy risk classification systems has been recommended by several scientific societies but radiologist’s adherence to these guidelines may vary. The authors aimed to analyze the quality of the information provided by the thyroid ultrasound report, to assess the malignancy risk of thyroid nodules, in Portugal.

**Methods:**

Multicenter and retrospective study, conducted in three of the five Portuguese NUTS2 corresponding to about 88.3% of the mainland population. We included 344 consecutive unselected participants aged ≥ 18 years who underwent thyroid ultrasonography in 2019. The description of six features of the dominant thyroid nodule was analyzed: maximum size, shape, margins, composition, echogenicity and echogenic foci. A utility score, including these six features, was used as an indicator of the report’s quality. A score of 4 was considered as a minimum value.

**Results:**

Maximum diameter was reported for all nodules. Shape, margins, composition, echogenicity and echogenic foci were reported in 8.1%, 25.0%, 76.5%, 53.2% and 20.9%, respectively. Only 21.8% of the nodules had a score ≥ 4. At least one of four suspicious features, including marked hypoechogenicity, microcalcifications, irregular margins and non-oval shape, was identified in 8.7% of the nodules. Cervical lymph nodes’ status was reported in 93% of the exams. The risk category was only reported in 7.8% of the participants.

**Conclusion:**

The adherence of Portuguese radiologists to a standardized reporting model and to an ultrasound-based malignancy risk stratification system is still low and has implications for the correct characterization of the malignancy risk of nodules and the decision to perform fine-needle aspiration biopsy.

## Background

Thyroid nodules are very common and its ultrasound (US) prevalence may reach 19–68%, depending on the studied population [1]. Despite their high prevalence, thyroid nodules have a relatively low risk of malignancy, around 3.1% and 4.7% in multinodular goiter and solitary nodule, respectively [2]. Thyroid cancer has a high worldwide incidence and generally has a good prognosis [3]. In Portugal, its incidence and mortality rate is respectively higher and lower than the world data [4]. In addition, the incidence of thyroid cancer has seen a global rise without a proportional increase in mortality [3–5]. The upward trend in the incidence rate of thyroid cancer has been mainly attributed to the diagnosis of asymptomatic cancers, namely microcarcinomas [6]. The increased detection of asymptomatic cancers can be caused by thyroid US “overscreening”, improving accessibility to health care services, and a trend towards an increase in the number of neck imaging studies and fine-needle aspiration biopsy (FNAB) procedures, In addition, the increase in the volume of thyroid surgery and changes in histopathological management guidelines of thyroid histological specimens may also contribute to these numbers [3, 5]. Overdiagnosis of thyroid cancer and its possible overtreatment may have important implications for patients and health care systems.

Clinical management guidelines for thyroid nodules have been changing over the last few years, namely with regard to a better characterization of the US-based malignancy risk and a trend towards a reduction in the number of nodules with indication for FNAB.

Similar to the Breast Imaging Reporting & Data System (BIRADS) classification used in breast US studies, the Thyroid Imaging Reporting & Data System (TIRADS) classification, an US-based malignancy risk stratification system for thyroid nodules, was introduced in 2009 by Horvath and colleagues [7]. In recent years, several scientific societies have proposed their own risk classifications. The US-based classification systems of the American Thyroid Association (ATA) [1], K-TIRADS of the Korean Society of Thyroid Radiology (KSThR) [8], ACR TI-RADS of the American College of Radiology (ACR) [9], and the EU-TIRADS of the European Thyroid Association (ETA) [10], have been widely used. The diagnostic performance comparison of these latest US-based malignancy risk-stratification systems has not shown clear differences between them [11–16]. EU-TIRADS has been widely accepted in Europe and was recently recommended in Portugal by the Thyroid Study Group (GET), of the Portuguese Society of Endocrinology, Diabetes and Metabolism (SPEDM) [17].

Taking into account limitations due to the particularities of each classification system, the cutoff points for FNAB are higher in the ACR TI-RADS and EU-TIRADS systems than in the ATA 2015 or K-TIRADS [16]. This leads to a decrease in the indications for FNAB [18]. Although ACR-TIRADS might have a slightly higher diagnostic accuracy than EU-TIRADS [15, 18], the use of a point scale for nodules classification may be excessively time-consuming in daily clinical practice. On the other hand, EU-TIRADS has shown to have a higher rate of inter-observer agreement than ACR TI-RADS in the classification and selection of nodules for FNAB [14]. Regardless of the classification system used, it is essential that the thyroid US reports, has the minimum necessary information for a correct risk stratification. Thus, the adoption of a standardized thyroid US report model that includes the use of a common lexicon [17, 19] and sufficient information [20, 21] may improve their quality [22]. US reports may, in many cases, not yet be adequate to this new reality [23]. In Portugal, the adherence of board-certified radiologists, who usually do thyroid US exams, to these classification systems seems to be quite low.

The GET intended to contribute to an improvement of the US reports through the development of a project called GET ECO. This initiative included the elaboration and publication of a position statement, based on the review of several international guidelines on the use of a common lexicon and US malignancy risk stratification systems [17]. This document was complemented with the development of an application called GET ECO (which can be consulted at https://get-eco.net), that uses the EU-TIRADS and ACR TI-RADS recommendations to support the drafting of a standardized report. In the context of this project, the authors set out to evaluate the quality of the information provided by the thyroid US report, for the characterization of the thyroid nodules malignancy risk according to EU-TIRADS classification in Portugal.

## Methods

The study was conducted in three of the five Portuguese NUTS2 level (Nomenclature of Territorial Units for Statistics 2), including 8,938,358 residents and corresponding to about 88.3% of the mainland population [24]. The following NUTS2 were included in this study: North (NUTS2 1), Center (NUTS2 2) and Lisbon and Tagus Valley (NUTS2 3). The proportion of the population residing in each NUTS2 was 41.4%, 25.7% and 32.9%, respectively.

This study was approved by the Administrative Council of the Portuguese Institute of Oncology of Coimbra Francisco Gentil, Portugal on June 5, 2019 and by its research ethics board on December 5, 2019 (Research work 40/2019).

A convenience, non-probability sampling method was used to select the endocrinologists to be invited for the study. In each NUTS2 region, three endocrinologists with proven experience in thyroid ultrasonography and accredited by GET were invited. The study participants were recruited from their patient lists. Patients aged 18 and over who had performed a thyroid ultrasonography between January 1st, and December 31st, 2019, were included. Most of the thyroid US exams had been ordered by a general practitioner. The endocrinologists involved in the study did not choose the radiologists who performed the thyroid ultrasonography. Patients with nodules smaller than 10 mm or with a past history of thyroid surgery or radioactive iodine treatment were excluded. All participants agreed to provide a copy of their thyroid US report and signed an informed consent. A sample size of 270 participants was estimated. Sample size was calculated considering a population estimate of 8,938,358 residents, a margin of error of 5%, a confidence level of 95% and a population proportion of 50%. Unselected participants, were consecutively included starting from the study start date (June 5, 2019) and until the required number of participants was reached. A copy of each participant's thyroid US report was made with the removal of any identification element. The study was carried out without the processing of personal data that would allow the identification of the participant, the radiologist or the institution that performed the exam. Personal health data of the participants regarding age, sex, NUTS2 of residence and some technical contents of the thyroid US report, were collected, recorded and processed. The reports of the thyroid US exams were retrospectively reviewed by the participating endocrinologists. In addition, all reports were submitted to a second independent review by another endocrinologist experienced in thyroid US. The EU-TIRADS classification was used to assess the malignancy risk [10].

The evaluation of the US report quality focused on the description of the thyroid nodule with the highest reported US-based risk, also called “dominant nodule”.

The thyroid US reports were analyzed to determine whether the description of the nodule features proposed by the EU-TIRADS classification had been included in the report. According to the EU-TIRADS system, the description of six features of the thyroid nodules was considered relevant: maximum diameter, shape, margins, composition, echogenicity and echogenic foci. These features allow to categorize the risk of malignancy of the nodules as well as the indications for FNAB. A utility score, including these six features, was used as an indicator of the report quality [23]. Each of these reported features was assigned as a point. Thus, each nodule had a score from 0 to 6. A score of 4 was considered as a minimum value for calculating the risk of malignancy according to the EU-TIRADS classification.

**Fig. 1:**
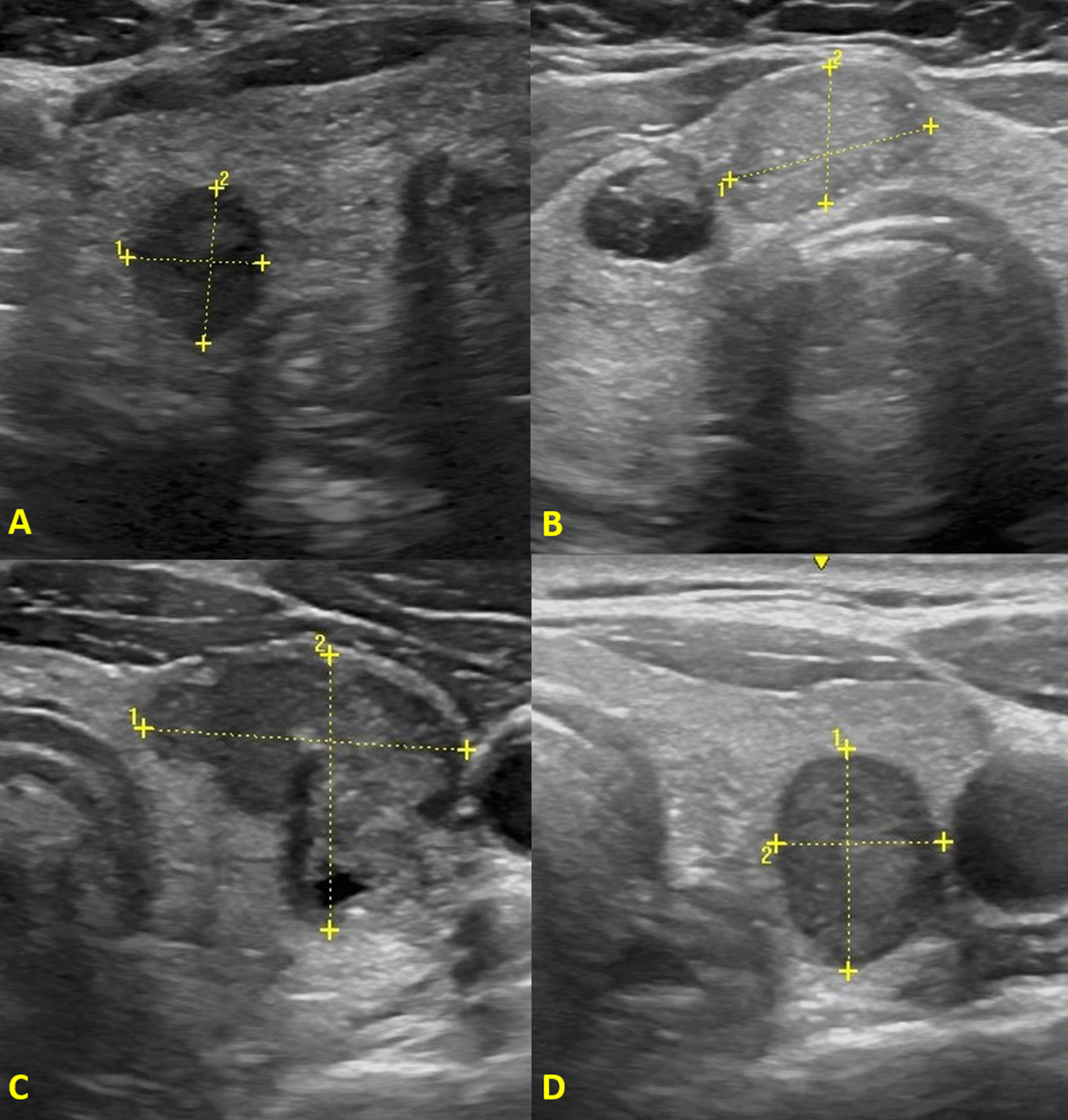
EU-TIRADS suspicious ultrasound features. **A** Marked hypoechogenicity, **B** microcalcifications, **C** irregular margins, **D** non-oval shape

The reference to the localization, the three-axis diameters, the volume and the vascularity of the thyroid nodule was also searched in the report. Furthermore, the reference to each of the four US features suspected of malignancy according to the EU-TIRADS classification was analyzed: marked hypoechogenicity, microcalcifications, irregular margins and non-oval shape (Fig. 1).

In addition, reference to a specific malignancy risk classification system was assessed. Finally, the number of non-dominant nodules with indication for FNAB was quantified: EU-TIRADS 3 nodules (low risk) larger than 20 mm; EU-TIRADS 4 nodules (intermediate risk) larger than 15 mm; EU-TIRADS 5 (high risk) nodules larger than 10 mm.

### Statistical Analysis

Descriptive statistic was used to report the study variables. Medians (interquartile range) and proportions were generated for continuous and categorical variables, respectively. Categorical variables were compared using the chi-square (χ^2^) test or Fisher’s exact test. The T Student’s test or the Mann–Whitney’s test were used to compare continuous variables. Statistical tests were 2-tailed. Significance for all statistic tests was set at *p* < 0.05. All statistical analyses were performed using IBM SPSS Statistic for Windows, version 22.0, Armonk, NY: IBM corp.

## Results

Thyroid US reports from 344 participants were analyzed. The median age was 59 years (interquartile range of 22) and there were no statistically significant differences in age distribution by sex (*p* = 0.623). The proportion of women was 90.1%. The location of the nodule was identified in most cases (99.7%). Maximum size was reported for all nodules. The median of the maximum size was 19 mm (interquartile range of 12). There were no sex statistically significant differences for maximum size (*p* = 0.370). There was a positive correlation between maximum size and age (*p* = 0.024). The proportion of nodules > 20 mm was 34.0% and 50.4% in participants aged ≤ 50 years and > 50 years, respectively (*p* = 0.006). Maximum size was greater than 15 mm and 20 mm in 240 (69.8%) and 156 (45.3%) nodules, respectively. Longitudinal, anteroposterior and transverse diameters were reported in 55 (16.0%), 44 (12.8%) and 29 (8.4%) of the nodules, respectively. The three-axis diameters were only reported in 27 (7.8%) nodules. Longitudinal, anteroposterior and transverse diameters were reported more often in NUTS2 Lisbon and Tagus Valley (24.9%, 23.1% and 14.2%, respectively) than in the other NUTS2. The nodule volume was calculated in only one case. Vascularity in thyroid nodules was reported in 20 (5.8%) of the participants. The proportion of nodules in which the five main US features, have been described, are presented in Table 1. Table 1 also shows the proportion of nodules in which reference was made to each of the four US features suspected of malignancy, according to the EU-TIRADS classification. The presence of at least one of these four suspicious features was identified in 30 (8.7%) nodules. A positive association (*p* = 0.004) was found between male sex and the presence of at least one of these factors. In addition, Table 1 shows the utility score assigned to the nodules (ranging from 0 to 6). Only 75 (21.8%) of the nodules had a utility score ≥ 4. Taking into account only the five main US features of the nodules, the reference to 0, 1, 2, 3, 4 or 5 of these features was identified in 32 (9.3%), 104 (31.7%), 119 (34.6%), 57 (16.6%), 21 (6.1%) and 6 (1.7%) of the nodules, respectively. There were statistically significant differences between the NUTS2 for composition (*p* = 0.012), echogenicity (*p* = 0.001), the utility score (*p* < 0.001), and score ≥ 4 (*p* = 0.006). Composition, echogenicity and a score ≥ 4 were reported more frequently in NUTS2 Lisbon and Tagus Valley than in other NUTS2. The risk category for malignancy was reported in only 27 (7.8%) nodules, predominantly in males (*p* = 0.010) and in NUTS2 North (*p* = 0.042, for differences between NUTS2). The nodules were classified according to the definitions ATA 2015, ACR (TI-RADS), ETA (EU-TIRADS) or others in 4, 12, 1 and 10 cases, respectively. Non-dominant nodules with indication for FNAB were described in 27 participants, with 14 (4.1%), 12 (3.5%) and 3 (0.9%) nodules in the low (> 20 mm), intermediate (> 15 mm) and high risk (> 10 mm) categories, respectively. Two participants had non-dominant nodules in two different categories. Mention on the cervical lymph nodes (central and lateral compartments) status was reported in 320 (93.0%) of the participants.

**Table 1:** Thyroid nodules ultrasound features

Ultrasound features	Total n (%)	NUTS2 North n (%)	NUTS2 Center n (%)	NUTS2 Lisbon n (%)	*p* value
Number of participants	344 (100%)	98 (28.5%)	77 (22.4%)	169 (49.1%)	–
Shape	28 (8.1%)	6 (6.1%)	11 (14.3%)	11 (6.5%)	0.081
Margins	86 (25.0%)	22 (22.4%)	13 (16.9%)	51 (30.2%)	0.065
Composition	263 (76.5%)	74 (75.5%)	50 (64.9%)	139 (82.2%)	0.012
Echogenicity	183 (53.2%)	38 (38.8%)	39 (50.6%)	106 (62.7%)	0.001
Echogenic foci	72 (20.9%)	20 (20.4%)	19 (24.7%)	33 (19.5%)	0.647
Marked hypoechogenicity	4 (1.2%)	0	2 (2.6%)	2 (1.2%)	0.282
Microcalcifications	20 (5.8%)	7 (7.1%)	8 (10.4%)	5 (3.0%)	0.056
Irregular margins	12 (3.5%)	4 (4.1%)	3 (3.9%)	5 (3.0%)	0.869
Non-oval shape	0	0	0	0	–
Score					
0	11 (3.2%)	0	9 (11.7%)	2 (1.2%)	< 0.001
1	39 (11.3%)	8 (8.2%)	8 (10.4%)	23 (13.6%)	
2	109 (31.7%)	44 (44.9%)	28 (36.4%)	37 (21.9%)	
3	110 (32.0%)	30 (30.6%)	22 (28.6%)	58 (34.3%)	
4	51 (14.8%)	11 (11.2%)	8 (10.4%)	32 (18.9%)	
5	18 (5.2%)	5 (5.1%)	2 (2.6%)	11 (6.5%)	
6	6 (1.7%)	0	0	6 (3.6%)	
≥ 4	75 (21.8%)	16 (16.3%)	10 (13.0%)	49 (29.0%)	0.005

## Discussion

Thyroid nodules are common, with an incidence 2–3 times higher in women than in men [25]. In our sample the men to women ratio was 0.1 but the median age was similar, suggesting that other factors may have contributed to this marked predominance of women. In Portugal, women's use of health services may be greater than in men which may imply greater consumption of health resources. In a recent study including 1339 patients, where the quality of the US report was assessed, the proportion of women was also high (85%) [23].

According to our results maximum size was reported in all nodules. As expected, a positive correlation was found between maximum size and age [26]. Previous studies also found a high reported maximum size feature [23, 27]. Maximum size is sometimes the only criterion used in daily practice to decide whether to perform FNAB or not. However, the size of the nodule alone has little value in assessing the risk of malignancy [28]. Maximum size, although not necessary for calculating the malignancy risk of the nodule, is a very important feature for the decision on the recommendation for FNAB according to the EU-TIRADS and other risk stratification systems [1, 8–10]. The three-axis diameters of the nodules were only mentioned in 7.8% of the participants and only in one case was the nodular volume calculated. Nodule diameters were more frequently reported in NUTS2 Lisbon and Tagus Valley than in the other NUTS2, suggesting some regional variability. In the follow-up of thyroid nodules, US growth criteria according to ATA 2015 and ACR TI-RADS (20% increase in at least two nodule diameters with a minimal increase of 2 mm or more than a 50% change in volume) requires information about the three-axis diameters or alternatively about the volume of the nodule. Furthermore, if the nodule shape is not mentioned, it is necessary to know the nodule diameters to calculate it. Given that the non-oval shape is a malignancy risk feature, high risk nodules may go unnoticed if information about the nodule shape is not available. According to our data, vascularity was only reported in 5.8% of participants. Intra-nodular vascularity has low predictive value, sensitivity and specificity in the diagnosis of malignancy [29, 30]. The EU-TIRADS guidelines states that, although the routine use of Doppler US is not recommended for US malignancy risk stratification it can be used to differentiate solid tissue from thick colloid or to enhance the detection of the limits of a nodule in an isoechogenic parenchyma [10]. In addition, the observation of a “spoke-and-wheel like” peripheral and central pattern of flow in a nodule can predict exceedingly bloody aspirates and non-diagnostic cytological findings [31].

The localization of the nodule is essential for its clinical integration and for a correct identification in future US exams and in preparation for FNAB or other diagnostic or therapeutic procedures. In the present study, the localization of the nodules was reported in the vast majority of cases. Other studies also confirm a high adherence by radiologists to the localization in the US report [23, 27].

Regarding the five main US features of the thyroid nodules, a very low report of shape, margins and echogenic foci was observed, with no significant differences between NUTS2. In contrast, the composition (76.5%) and echogenicity (53.2%) have often been reported, with significant differences between NUTS2. At NUTS2 Lisbon and Tagus Valley, the composition and the echogenicity features were reported more frequently than in the remaining NUTS2. In a previous study on the quality of US exams there was also a low report of the margins (8%) and echogenic foci (14%) and no reference to the shape [22]. Composition and echogenicity were mentioned in 72% and 59% of the reports, respectively. In another study the composition (64.0%) and echogenicity (43.8%) features were also more reported than margins (11.0%), echogenic foci (35.1%) and shape (3.9%) features [23]. A recent Canadian study also showed a higher report of composition (64.0%) and echogenicity (42.0%) than shape (2.0%), echogenic foci (33.0%) and margins (22.0%) features [27].

Dominant nodules classified as EU-TIRADS 5 (with at least one suspicious feature) were reported in 8.7% of participants. In a previous study, which included 1029 nodules 7.4% of the nodules were categorized as EU-TIRADS 5 [32]. In addition the same study showed the presence of marked hypoechogenicity, microcalcifications, irregular margins and non-oval shape in 1.5%, 3.3%, 1.4% and 2.8% of nodules. In another study in which the quality of US reports was analyzed, non-oval shape and marked hypoechogenicity were never reported [22]. In addition, microcalcifications and irregular margin were only reported in 3% and 1% of nodules, respectively. Compared to our results, microcalcifications and irregular margins were less reported in those studies. Our data confirms the low non-oval shape US report. According to our results, malignancy risk features were reported more often in men than in women. Radiologists may be more careful in men due to their increased risk of differentiated thyroid carcinoma (DTC). In fact, men with nodular thyroid disease undergoing FNAB have a higher risk of DTC than women, although the prevalence of the disease and the absolute number of cancers is higher in women [33]. In addition, men with DTC may have a more advanced disease at the time of diagnosis, although male sex is not an independent prognostic factor in itself [34].

According to our results, the utility score of 6 was observed in only 1.7% of the nodules. In addition, only 21.8% of the nodules had a score ≥ 4. Our data is slightly better compared to the results of a previous study in which the score of 6 and the score ≥ 4 were identified in only 0.4% and 13.7%, respectively [23].

Most of the reports (92.2%) did not refer a risk category. The risk category was reported more often in men than in women. As mentioned above, this finding may be due to greater attention from radiologists in men due to its increased risk. The reference to the EU-TIRADS classification system was made in only one participant. These results document the low adherence in Portugal, namely in NUTS2 Center and Lisbon and Tagus Valley to these classification systems in clinical practice. In addition, and although the European-based EU-TIRADS classification, developed by ETA, was published in 2017, its use by Portuguese radiologists is residual. Other studies have shown greater adherence by radiologists to the US-based risk stratification systems. A Canadian study showed that 29.9% of reports cited a risk stratification system [27].

The description of non-dominant nodules with criteria for FNAB was made in only 8.4% of the participants. These results suggest that, in most cases, the report was focused on just one nodule considered to be "dominant".

According to our results, only 7.0% of reports did not mention the presence or absence of cervical lymph nodes. These results are better than those found in another study that included 136 participants and showed that 74% of the US examinations did not mention lymph node status at all [35].

According to several scientific societies, the use of a thyroid imaging and reporting data system is very useful for physicians in their clinical practice [1, 10]. The use of US pattern categories, which include several individual sonographic features, when compared to individual US characteristics, increase the interobserver report reproducibility. In addition, these systems offer a simple communication of results, facilitating decision-making regarding the indication for diagnostic FNAB. Taking into account our results, most reports did not allow the establishment of the nodules’ malignancy risk and US features of aggressive thyroid cancers, thus preventing an informed decision on the recommendation of diagnostic FNAB. It is therefore urgent to make radiologists aware of the need to adhere to these US-based risk stratification systems. Studies like this can contribute to the improvement of clinical practice and to a reduction of unnecessary diagnostic interventions. Finally, there are some strengths and limitations of this study that should be mentioned. It is a multicenter one involving participants from the three largest NUTS2 in mainland Portugal. The thyroid US exams were performed by radiologists in their routine clinical practice as part of their activity in private radiology centers and not in specialized centers. In this way our results show the real life clinical practice. In this study, a non-probabilistic sampling method was used, with convenience selection of participating endocrinologists. For this reason, biases with interference in the representativeness of the results cannot be excluded. However, unselected participants were consecutively included. Furthermore, most of the thyroid US exams had been ordered by a general practitioner and endocrinologists involved in the study did not choose the radiologists who performed the exams. It should also be noted that, despite three endocrinologists from each NUTS2 region being selected, there was no further sampling stratification, namely with regard to the volume of exams performed by each radiologist, as it was not possible to access this information. The retrospective nature of this study did not prevent access to the full content of the reports. The final sample size was determined by the number of participants included within the approved study time. NUTS2 Center did not reach the expected number of participants. In contrast, NUTS2 Lisbon and Tagus Valley included more participants than expected. It was assumed that the non-reference in the report to a nodule feature did not mean the absence of that finding. In some cases, this may have been the radiologist's intention, but the failure to include that information in the report prevented the risk assessment of the nodule.

## Conclusions

The adherence of Portuguese radiologists to a standardized reporting model and to US-based malignancy risk stratification systems is still low. Our results also confirm that, when one of these systems was not used, it was not possible, in most cases, to establish the malignancy risk of the nodules. This study highlights the importance of using these classification systems in the characterization of the nodules’ risk and in supporting the informed decision to perform FNAB, thus contributing to a better selection of nodules and reducing unnecessary diagnostic procedures.

## Data Availability

The datasets used and/or analysed during the current study are available from the corresponding author on reasonable request.

## Abbreviations

US: Ultrasound
FNAB: Fine-needle aspiration biopsy
BIRADS: Breast Imaging Reporting & Data System
TIRADS: Thyroid Imaging Reporting & Data System
ATA: American Thyroid Association
KSThR: Korean Society of Thyroid Radiology
ACR: American College of Radiology
ETA: European Thyroid Association
GET: Thyroid Study Group (Grupo de Estudo da Tiroide)
SPEDM: Portuguese Society of Endocrinology, Diabetes and Metabolism (Sociedade Portuguesa de Endocrinologia, Diabetes e Metabolismo)
NUTS2: Nomenclature of Territorial Units for Statistics 2
DTC: Differentiated thyroid carcinoma
